# RanBP9/TSSC3 complex cooperates to suppress anoikis resistance and metastasis via inhibiting Src-mediated Akt signaling in osteosarcoma

**DOI:** 10.1038/cddis.2016.436

**Published:** 2016-12-29

**Authors:** Huanzi Dai, Yang-Fan Lv, Guang-Ning Yan, Gang Meng, Xi Zhang, Qiao-Nan Guo

**Affiliations:** 1 Department of Pathology, Xinqiao Hospital, The Third Military Medical University, Chongqing, People's Republic of China; 2 Department of Nephrology, Daping Hospital, Third Military Medical University, Chongqing, People's Republic of China; 3 Department of Pathology, Southwest Hospital, The Third Military Medical University, Chongqing, People's Republic of China

## Abstract

Suppression of anoikis is a prerequisite for tumor cell metastasis, which is correlated with chemoresistance and poor prognosis. We characterized a novel interaction between RanBP9 SPRY domain and TSSC3 PH domain by which RanBP9/TSSC3 complex exerts transcription and post-translation regulation in osteosarcoma. RanBP9/TSSC3 complex was inversely correlated with a highly anoikis-resistant phenotype in osteosarcoma cells and metastasis in human osteosarcoma. RanBP9 cooperated with TSSC3 to inhibit anchorage-independent growth and to promote anoikis *in vitro* and suppress lung metastasis *in vivo*. Moreover, RanBP9 SPRY domain was required for RanBP9/TSSC3 complex-mediated anoikis resistance. Mechanistically, RanBP9 formed a ternary complex with TSSC3 and Src to scaffold this interaction, which suppressed both Src and Src-dependent Akt pathway activations and facilitated mitochondrial-associated anoikis. Collectively, the newly identified RanBP9/TSSC3 complex cooperatively suppress metastasis via downregulation of Src-dependent Akt pathway to expedite mitochondrial-associated anoikis. This study provides a biological basis for exploring the therapeutic significance of dual targeting of RanBP9 and TSSC3 in osteosarcoma.

The metastatic cascade involves a series of discrete steps including dissociation of cancer cells from the primary site, intravasation and survival in the circulation, and extravasation and growth in a distant target organ.^1, 2^ As a barrier to metastasis, loss of inappropriate cell adhesion normally induces apoptosis via a process termed anoikis.^3^ Anoikis prevents adherent cells from survival in suspension or detached cells to re-adhere to new matrices and grow in wrong locations. Suppression of anoikis is likely to be a prerequisite for metastasis, and cancer cells that acquire malignant potential develop mechanisms to resist anoikis and can therefore participate in the metastatic cascade.^4, 5, 6^ Therefore, restoration of anoikis sensitivity may limit the uncontrolled spread of metastatic tumors.

Osteosarcoma, the most common primary malignant tumor of bone, is highly aggressive and metastatic with lung metastasis as the major factor to affect therapy and prognosis. The molecular mechanisms underlying anoikis resistance and metastasis in osteosarcoma are poorly understood, although several investigations have provided some insight.^7^ Using microarray analysis, we previously reported that tumor-suppressing STF cDNA 3 (*TSSC3)* was related to malignant transformation of human osteoblast hFOB1.19 cells.^8^ *TSSC3*, the first apoptosis-related gene that has been shown to be imprinted and expressed from maternal alleles during normal development, is located within the 11p15 tumor-suppressor region and is downregulated in several human cancers including osteosarcoma.^9, 10, 11, 12, 13, 14, 15, 16, 17^ This prompted us to explore whether TSSC3 has a role in anoikis resistance and metastasis in osteosarcoma and investigate the underlying mechanisms. RanBP9 is widely expressed in most tissues and cell lines, and contains multiple conserved domains that provide potential protein–protein interaction sites. RanBP9, a crucial component of numerous multi-protein complexes, modulates and/or interacts with a wide range of proteins as a protein stabilizer or regulator of transcriptional activity, and thereby regulates diverse cellular functions including cell proliferation, differentiation, apoptosis, cell cycle progression, adhesion and migration.^18, 19, 20, 21^

Src family kinases are essential elements of anoikis resistance signaling pathways. Activated Src leads to recruitment of PI3K and subsequent production of the second messenger phosphatidylinositol (3,4,5)-triphosphate (PIP3), which further recruits and activates its downstream target protein kinase Akt. Activated Akt suppresses anoikis resistance through phosphorylation of the pro-apoptotic proteins Bad and Bim and release of the anti-apoptotic protein Bcl-2, which prevents mitochondrial release of cytochrome c to inhibit the intrinsic cell death pathways. Both Src and Akt activations are frequently observed in a variety of epithelial and non-epithelial cancers that are resistant to anoikis.^7, 22, 23, 24, 25, 26^

Here, we applied yeast two-hybrid screening of a human fetal brain library using full-length *TSSC3* cDNA as bait to identify RanBP9 as a novel putative binding partner for TSSC3. Mechanistically, we characterized the novel functional interaction among RanBP9 and TSSC3 as well as Src, and demonstrated that this complex cooperated to regulate anoikis resistance, migration, invasion and metastasis in osteosarcoma.

## Results

### Identification of RanBP9 as a novel TSSC3-interacting protein in human osteosarcoma cell lines

**Figure 1:**
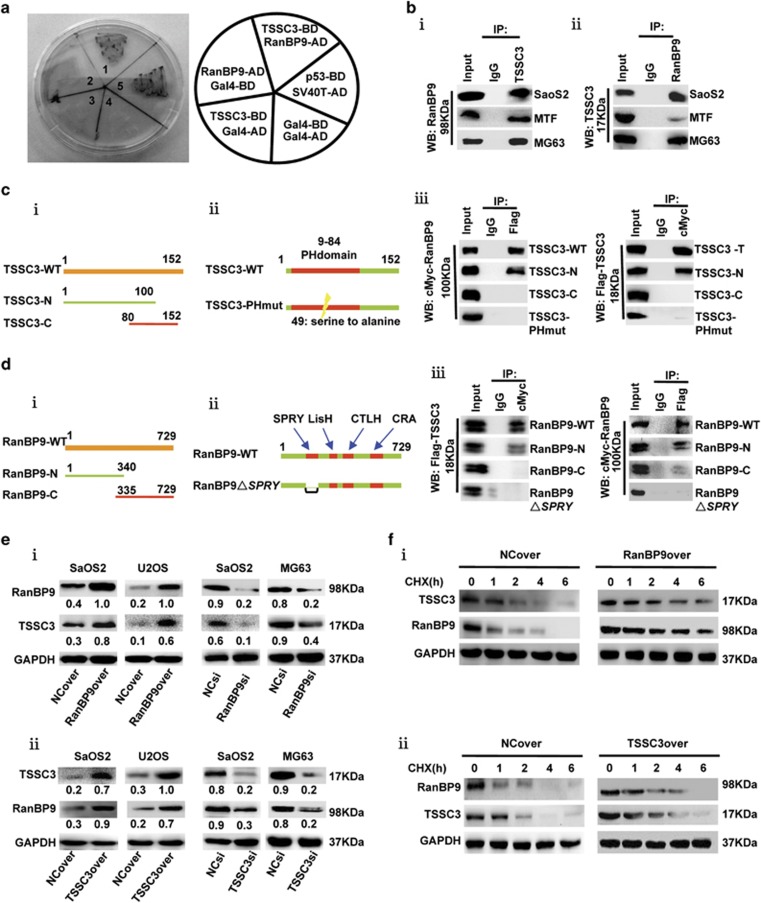
RanBP9 interacts with TSSC3 via post-translational mechanism. (**a**) Yeast strain Y190 strain was co-transformed with the indicated binding domain (BD) plasmids and activation domain (AD) plasmids. Co-expression of BD-TSSC3 and AD-RanBP9 induced formation of blue colonies on SD/-Trp/-Leu, similarly to positive control cells expressing murine p53 and SV-40 large T-antigen. Gal4-BD and Gal4-AD were used as negative controls. (**b**) Confirmation of the interaction between endogenous RanBP9 and TSSC3 in osteosarcoma cells. Co-immunoprecipitation assays of whole-cell lysates using anti-TSSC3, or nonspecific IgG and probed with anti-RanBP9 (i), or anti-RanBP9 and probed with anti-TSSC3 (ii). Input samples indicate 10% of pre-immunoprecipitated samples. (**c**) RanBP9 interacts with TSSC3 PH domain. (i) Schematic illustration of the TSSC3 N- and C-terminal constructs and (ii) PH domain-mutant construct (TSSC3-PHmut, the 49th amino acids serine was mutated to alanine). (iii) Immunoprecipitation assays of 293 T cells co-transfected with the indicated constructs using either anti-Flag or anti-cMyc, followed by immunoblot with anti-cMyc or anti-Flag, respectively. (**d**) TSSC3 interacts with RanBP9 SPRY domain. (i) Schematic illustration of the RanBP9 N- and C-terminal constructs, and (ii) SPRY domain-deleted construct of RanBP9 (RanBP9△SPRY, △aa 212–333). (iii) Immunoprecipitation assays of 293 T cells co-transfected with the indicated constructs using either anti-Flag or anti-cMyc, followed by immunoblot with anti-cMyc or anti-Flag, respectively. (**e**) Western blot analysis of TSSC3 and RanBP9 in the indicated RanBP9-overexpressing and RanBP9-knockdown (i) or TSSC3-overexpressing and TSSC3-knockdown (ii) cells. Western blot values were normalized to GAPDH. (**f**) (i) RanBP9 increases the half-life of TSSC3. SaOS2 cells expressing empty vector (NC) or RanBP9 were treated with cycloheximide (CHX, 100 *μ*g/ml) for the indicated times and subjected to immunoblotting as indicated. (ii) TSSC3 increases the half-life of RanBP9. SaOS2 cells expressing empty vector or TSSC3 were processed as described in Figures 1f (i)

We applied yeast two-hybrid screening of a human fetal brain library by using full-length *TSSC3* cDNA as bait to isolate 10 positive clones, of which 5 encoded RanBP9 protein fragments (Supplementary Table S1), suggesting that RanBP9 is a novel putative binding partner for TSSC3, which was further validated by yeast mating experiments (Figure 1a). Co-immunoprecipitation of SaOS2, MTF and MG63 cell lysates, where both endogenous RanBP9 and TSSC3 protein are expressed, confirmed the formation of a complex between RanBP9 and TSSC3 in osteosarcoma cells (Figure 1b).

### Characterization of the interaction between RanBP9 and TSSC3

Immunoprecipitation assays using both N-terminal and C-terminal constructs of TSSC3 demonstrated that RanBP9 bound to the N-terminal region (amino acids 1–100) of TSSC3 (Figure 1c), which contains a pleckstrin homology (PH) domain. RanBP9 was co-immunoprecipitated with wild-type TSSC3 but not PH domain-mutated TSSC3, confirming that the PH domain is required for the interaction between RanBP9 and TSSC3 (Figure 1c).

Immunoprecipitation assays using both N-terminal and C-terminal constructs of RanBP9 revealed that TSSC3 bound to the N-terminal region of RanBP9 (Figure 1d). RanBP9 possesses a modular domain structure comprising SPRY, LISH, CTLH and CRA domains (Figure 1d(ii)), and SPRY is the only domain in the N-terminal region of RanBP9. Deletion of SPRY domain significantly reduced the ability of RanBP9 to interact with TSSC3 (Figure 1d). These results indicate that both SPRY domain of RanBP9 and PH domain of TSSC3 are required for the interaction between these proteins.

### RanBP9 and TSSC3 interact via both transcriptional and post-translational mechanisms

To investigate the significance of the interaction between RanBP9 and TSSC3, we generated SaOS2, U2OS and MG63 osteosarcoma cell lines that stably overexpressed RanBP9 or TSSC3 (RanBP9over or TSSC3over) or stably knocked down *RanBP9* or TSSC3 (RanBP9si or TSSC3si), as well as the corresponding control cells (NCover or NCsi). RanBP9 functions as a protein stabilizer.^18^ Overexpression of RanBP9 increased whereas knockdown of *RanBP9* reduced endogenous TSSC3 protein expression (Figure 1e(i)). In the cells treated with the protein synthesis inhibitor cycloheximide to evaluate protein degradation, the degradation of TSSC3 was greatly reduced in the presence of RanBP9 (Figures 1f(i)), further suggesting that RanBP9 stabilizes TSSC3. Intriguingly, overexpression of TSSC3 increased but knockdown of *TSSC3* decreased endogenous RanBP9 protein abundance, and also markedly increased the half-life of endogenous RanBP9 (Figures 1e and f(ii)). In addition, RanBP9 and TSSC3 could also alter the expression of each other at the transcriptional level (Supplementary Figures S1a and b). And the luciferase reporter promoter assay showed that RanBP9 overexpression increased the promoter activity of TSSC3 and RanBP9 downregulation suppressed the promoter activity in SaOS2 cells, whereas TSSC3 upregulation increased the promoter activity of RanBP9 and TSSC3 downregulation suppressed the promoter activity (Supplementary Figure S1c). These results suggest that RanBP9 and TSSC3 interact via both transcriptional and post-translational mechanism.

### Loss of RanBP9 and TSSC3 promotes a highly anoikis-resistant phenotype in osteosarcoma cell lines

TSSC3 is reduced in human osteosarcoma cell lines.^14, 15, 16, 17^ We observed that both RanBP9 and TSSC3 mRNA and protein expressions were significantly decreased not only in the malignant transformed hFOB1.19 (MTF) osteoblasts as compared with hFOB1.19 osteoblasts, but also in the highly metastatic MTF cell lines (cell lines derived from high-grade osteosarcoma) as compared with the less-metastatic SaOS2, U2OS or MG63 cell line (a cell line derived from low-grade osteosarcoma) (Supplementary Figures S2a and b). Immunofluorescent analysis demonstrated that RanBP9 was localized in the nucleus and cytoplasm, whereas TSSC3 was primarily localized in the cytoplasm (Supplementary Figure S2c).

Parental SaOS2 cells underwent anoikis when attachment to the extracellular cell matrix was prevented by culture under suspension conditions in the ultralow attachment culture plates, accompanied by enhancement of cell death in a time-dependent manner (Supplementary Figure S3a). As culture continued, the small, loose cell aggregates that survived in the ultralow attachment culture plates became large and compact (Supplementary Figure S3b), suggesting that the cells underwent increased anoikis resistance. Next, we sorted surviving anoikis-resistant cells (Annexin V^-^/7AAD^-^ cells) by flow cytometry after culture in either adherent or suspension conditions. The mRNA level and protein abundance of RanBP9 or TSSC3 were both downregulated in a time-dependent manner in the parental SaOS2 and MG63 cells that survived under suspension conditions as compared with adherent conditions (Supplementary Figures S3c and d). These results indicate that reduction in RanBP9 or TSSC3 is correlated with the anoikis resistance in osteosarcoma cells.

### Loss of RanBP9 and TSSC3 correlates with metastasis in human osteosarcoma

We performed immunohistochemical staining of human osteosarcoma tissues to investigate the relevance of RanBP9 and TSSC3 *in vivo*, and found that expressions of both RanBP9 and TSSC3 (Supplementary Figure S4) were significantly associated with lung metastasis, local recurrence and Enneking stage, but not age, sex, primary location, tumor size and pathological subtype (Table 1). The positive rates of RanBP9 and TSSC3 were 23% (3/13) and 38% (5/13), respectively, in osteosarcoma with metastasis, as well as 91% (61/67) and 88% (59/67), respectively, in osteosarcoma without metastasis. High-grade disease is currently the best prognostic indicator of metastasis, which, in turn, is the most informative indicator of poor survival.^27^ Overall, all cases with negative RanBP9 or TSSC3 expression were high-grade osteosarcoma, indicating that RanBP9 and TSSC3 are reduced in high-grade osteosarcoma. A positive correlation was observed between RanBP9 and TSSC3 expression (*P*<0.001, Supplementary Table S2). Ezrin is the only other protein associated with metastasis in osteosarcoma,^28^ and this relationship was confirmed in this study (*P*<0.001, Supplementary Table S3), with negative correlations observed between the expression of Ezrin and RanBP9 or TSSC3 (*P*<0.001, Supplementary Table S4). Taken together, we inferred that loss of RanBP9/TSSC3 expression may be associated with lung metastasis in human osteosarcoma.

### The RanBP9/TSSC3 complex inhibits anchorage-independent cell growth and sensitizes osteosarcoma cells to anoikis

**Figure 2:**
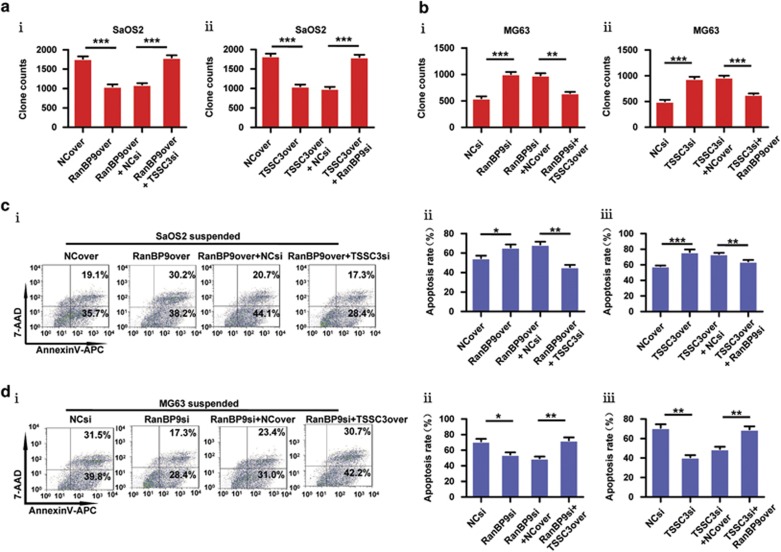
RanBP9 and TSSC3 function cooperatively to inhibit anchorage-independent cell growth and sensitize osteosarcoma cells to anoikis. SaOS2 or MG63 cells were stably transfected with the indicated constructs. (**a** and **b**) Soft agar assays. SaOS2 (**a**) or MG63 (**b**) cells (20 000) were seeded, cultured for 10 days in media supplemented with 10% FBS and the number of colonies was determined. (**c**, and **d**) Apoptosis assays. SaOS2 (**c**) or MG63 (**d**) cells were cultured under suspension conditions and subjected to flow cytometric analysis. Representative flow cytometric analyses (i) and quantification (ii and iii) of the percentages of early apoptotic cells (Annexin V^+^, PI^-^) and late apoptotic or necrotic cells (Annexin V^+^/PI^+^). Note: all values are mean±S.D. of three independent experiments each performed in triplicate; *P<0.05, **P<0.01, ***P<0.001

Anchorage-independent growth and anoikis resistance are characteristics of malignant cells.^29^ We used gain-and-loss functional approaches in the SaOS2 cell line with relatively low expression of RanBP9 or TSSC3, and loss-and-gain functional approaches in the MG63 cell line with relatively high expression of RanBP9 or TSSC3 to assess the role of RanBP9/TSSC3 complex in the anoikis-resistant phenotype (Supplementary Figure S5). Overexpression of RanBP9 not only abrogated cell growth under anchorage-independent conditions but also exacerbated cell death in suspension culture, which were significantly reversed by simultaneous knockdown of *TSSC3* (Figure 2a(i); Figures 2c(i and ii)). In contrast, RanBP9-knockdown MG63 cells produced more colonies and underwent less cell death under anchorage-independent conditions, and these affects were inverted by overexpression of TSSC3 (Figure 2b(i); Figure 2d(i and ii)). As expected, knockdown or overexpression of RanBP9 reversed the effects on anchorage-independent growth and anoikis resistance induced by overexpression or knockdown of TSSC3, respectively (Figure 2a(ii); Figure 2c(iii); Figure 2b(ii); Figure 2d(iii)). In addition, we also comfirmed RanBP9/TSSC3 complex could regulate apoptosis under adherent culture condition (Supplementary Figure S6). Taken together, we conclude that the functional interaction between RanBP9 and TSSC3 regulates anoikis resistance in osteosarcoma.

### The RanBP9/TSSC3 complex suppresses lung metastasis

**Figure 3:**
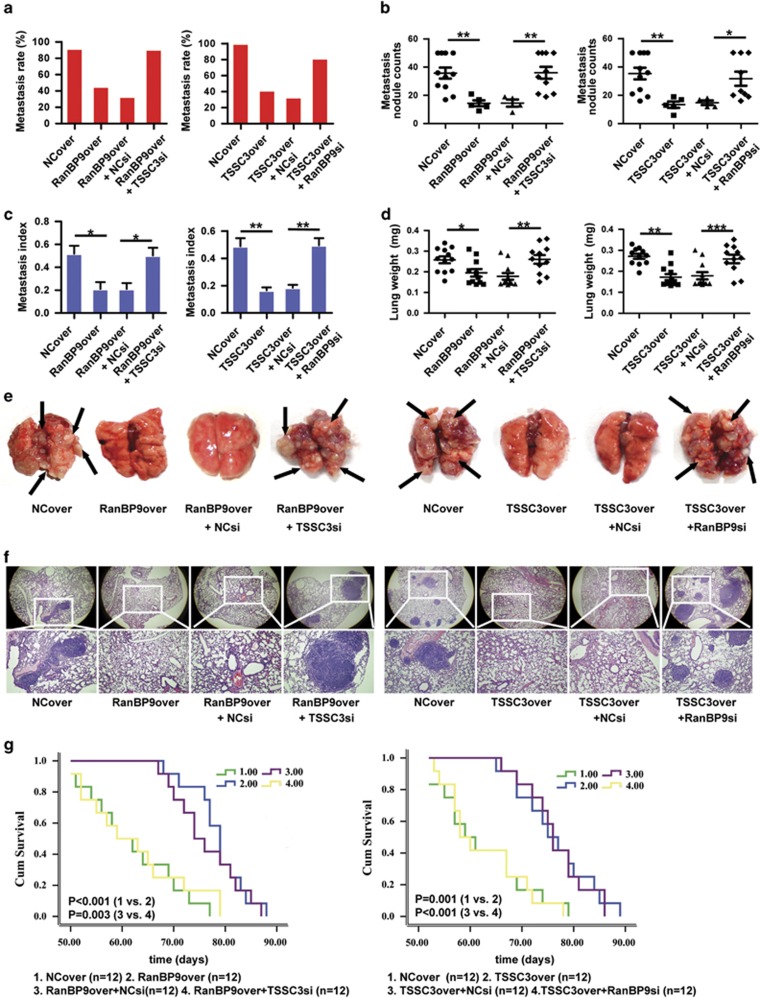
RanBP9 and TSSC3 function cooperatively to suppress lung metastasis. MTF cells were transfected as indicated and injected into the tail vein of athymic nude mice to establish an *in vivo* model of lung metastasis. The animals were killed 63 days later. (**a**) Percentage of mice bearing lung metastases. (**b**) Numbers of visible lung metastatic nodules on the surface of the lung. (**c**) Lung metastasis index (ratio of tumor area to the total tumor and lung area). (**d**) Mean weight of the lungs. All values in (**a**–**d**) are mean±s.d. of the 11 or 12 mice per group. * *P*<0.05, **P<0.01, ***P<0.001. (**e**) Representative macroscopic images of the lungs. Arrows indicate metastatic lesions. (**f**) Representative hematoxylin and eosin-stained images of the lungs shown in (**e**). (**g**) Kaplan–Meier overall survival curves

To confirm whether the RanBP9/TSSC3 complex suppresses metastasis in osteosarcoma, we established an *in vivo* lung metastasis model by injecting transfected MTF cells into the tail vein of nude mice. Overexpression of RanBP9 or TSSC3 significantly reduced the ability of the cells to establish lung metastases, as reflected by the incidence, number, size and weight of the metastatic nodules. However, knockdown of *TSSC3* or *RanBP9* restored the ability of RanBP9- or TSSC3-overexpressing cells, respectively, to establish metastases (Figures 3a and f). As expected, the lung nodules were negative for CK18 but positive for BMP and vimentin, indicating their mesenchymal origin (Supplementary Figure S7). Metastasis is a major prognostic factor in osteosarcoma.^7^ Overexpression of RanBP9 or TSSC3 was associated with favorable survival (90 days as end point) as compared with the control mice of which all died before day 80. Consistent with the findings mentioned above, knockdown of *TSSC3* or *RanBP9* significantly attenuated the survival advantage observed in mice injected with RanBP9- or TSSC3-overexpressing cells, respectively (Figure 3g). These *in vivo* results corroborate our *in vitro* findings to highlight a significant functional role for the RanBP9/TSSC3 complex in the regulation of lung metastasis in osteosarcoma.

### Crucial involvement of Src in RanBP9/TSSC3 complex-suppressed anoikis resistance

**Figure 4:**
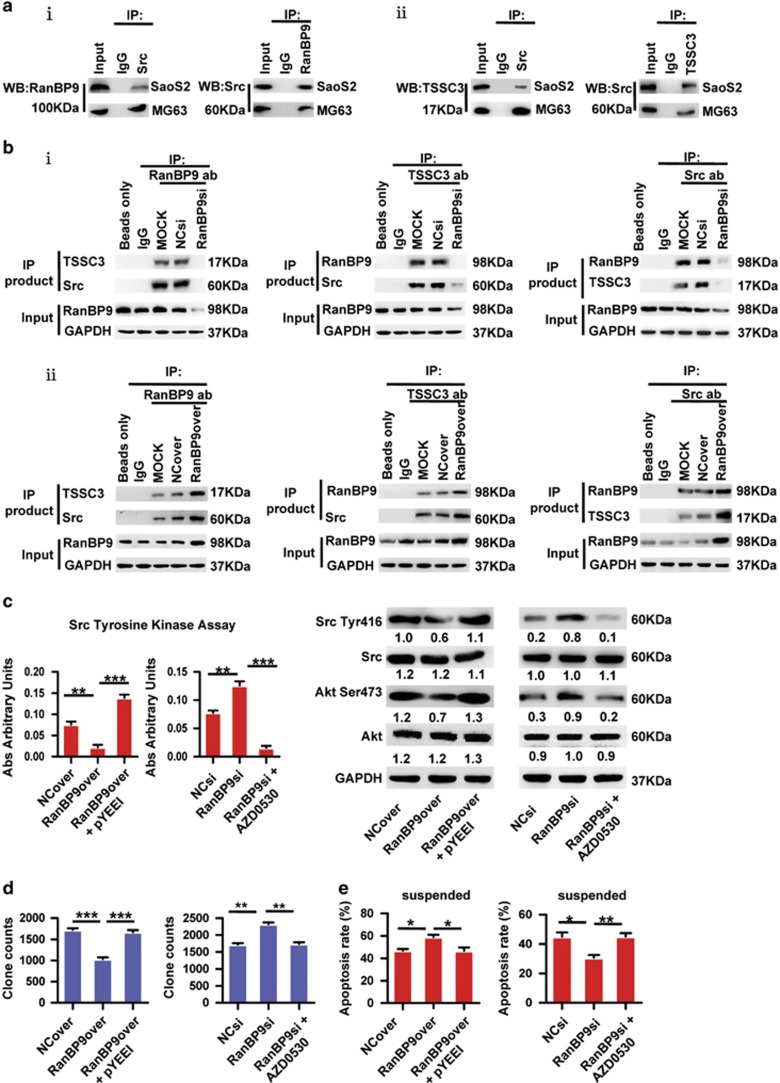
Crucial involvement of Src in RanBP9/TSSC3 complex-mediated anoikis resistance. (**a**) Confirmation of the formation of RanBP9/Src and TSSC3/Src complexes in osteosarcoma cells. Cell lysates were immunoprecipitated with anti-Src or anti-RanBP9 and immunoblotted with anti-RanBP9 or anti-Src (i), or immunoprecipitated with anti-Src or anti-TSSC3 and immunoblotted with anti-TSSC3 or anti-Src (ii). (**b**) The RanBP9/TSSC3/Src interaction depends on RanBP9 expression. Co-immunoprecipitation experiments in *RanBP9*-knockdown (i) or RanBP9-overexpressing (ii) SaOS2 cells using anti-RanBP9, anti-TSSC3 or anti-Src. Nonspecific IgG or no antibody (beads only) precipitates were loaded as negative controls. Blots were probed with antibodies against TSSC3 and Src, RanBP9 and Src, or RanBP9 and TSSC3. (**c**) Src kinase activity is modulated by RanBP9 in SaOS2 cells. RanBP9-overexpressing cells were treated with or without the Src activator pYEEI and *RanBP9*-knockdown cells were treated with or without the Src inhibitor AZD0530. Left, Src tyrosine kinase assay. Right, western blot analysis of Src and Akt expression and phosphorylation. Western blot values of the Src Tyr^416^, Src and Akt, Akt Ser^473^ bands were was normalized to GAPDH. (**d** and **e**) Src kinase activity modulates the anchorage-independent growth and anoikis resistance of SaOS2 cells. Soft agar colony formation assay (**d**) and flow cytometric analysis of apoptosis (**e**) for the indicated stable transfectants in which RanBP9 was overexpressed or knocked down and cultured in suspension in the presence or absence of Src activator pYEEI (100 *μ*mol/l) or Src inhibitor AZD0530 (3 *μ*mol/l). Note: all values are mean±s.d. of three independent experiments each performed in triplicate; *P<0.05, **P<0.01, ***P<0.001

Src kinase is not only highly activated in various cancers but is also involved in the progression of osteosarcoma.^30, 31^ We evaluated the role of Src kinase as a possible mediator of RanBP9/TSSC3 complex function. Co-immunoprecipitation assays indicated RanBP9, TSSC3 and Src ternary complex formation in SaOS2 cells (Figure 4a), and knockdown or overexpression of RanBP9 significantly attenuated or augmented the interactions between RanBP9 and TSSC3, RanBP9 and Src, and TSSC3 and Src (Figure 4b). Thus, we addressed whether Src kinase activity was mediated by the RanBP9/TSSC3/Src ternary complex. Overexpression of RanBP9 reduced Src enzymatic activity, Src Tyr^416^, Akt Ser^473^, anchorage-independent growth and anoikis resistance, whereas knockdown of *RanBP9* had the opposite effects (Figures 4c–e; Supplementary Figure S8a). Exposure of RanBP9-overexpressing cells to the Src activator pYEEI restored Src kinase activity, Src Tyr^416^, Akt Ser^473^and anoikis resistance whereas the Src inhibitor AZD0530 abrogated Src activation, Src Tyr^416^, Akt Ser^473^ and anoikis resistance induced by knockdown of *RanBP9* (Figures 4c–e; Supplementary Figure S8a), implying that Src kinase activity is critical for RanBP9/TSSC3 complex-suppressed anoikis resistance.

### The RanBP9/TSSC3 complex facilitates mitochondrial-associated anoikis via downregulating the Src/Akt pathway

**Figure 5:**
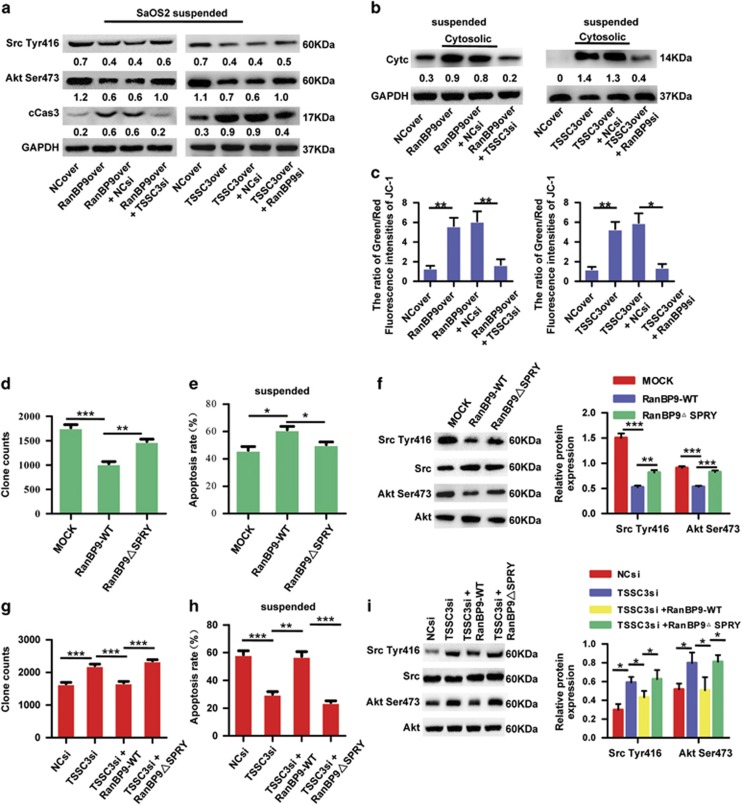
The RanBP9/TSSC3 complex regulates mitochondrial-associated anoikis via negatively regulating the Src/Akt pathway and the RanBP9 SPRY domain is required for RanBP9/TSSC3 complex-mediated resistance to anoikis. (**a**) The RanBP9/TSSC3 complex regulates anoikis. Western blot analysis of Src Tyr^418^, Akt Ser^473^ and cleaved caspase-3 expression in stable SaOS2 cells transfected as indicated and cultured under suspension conditions. Western blot values were normalized to GAPDH. (**b**) Cytochrome c release assay. SaOS2 cells were stably transfected as indicated and cultured under suspension. Western blot analysis demonstrated the release of (Cyt c) to the cytosol. Western blot values were normalized to GAPDH. (**c**) Mitochondrial transmembrane potential assay. SaOS2 cells were stably transfected as indicated and cultured under suspension. The ratio of green/red fluorescence intensities of JC-1 were calculated from JC-1 staining of the indicated SaOS2 transfectants. (**d-f**) Deletion of the SPRY domain reduced the ability of RanBP9 to cooperate with endogenous TSSC3 and attenuated anoikis. SaOS2 cells transfected with MOCK, RanBP9-WT or RanBP9△SPRY were subjected to the soft agar assay (**d**), flow cytometric analysis after suspension culture (**e**) or western blotting analysis and using anti-Src Tyr^416^, anti-Src, anti-Akt Ser^473^ and anti-Akt (**f**). Western blot values of the Src Tyr^416^ and Akt Ser^473^ bands were normalized to the corresponding Src and Akt bands. (**g-i**) Deletion of the SPRY domain SPRY of RanBP9 does not reverse TSSC3si-enhanced anoikis resistance. SaOS2 cells co-transfected with the TSSC3si/NCsi together with wild-type RanBP9 or RanBP9△SPRY were subjected to the experiments described in **d**, **e** and **f**. Western blot values of the Src Tyr^416^ and Akt Ser^473^ bands were normalized to the corresponding Src and Akt bands. Note: all values are mean±s.d. of three independent experiments each performed in triplicate; *P<0.05, **P<0.01, ***P<0.001

Both RanBP9 and TSSC3 are implicated in mitochondrial-mediated apoptosis.^14, 15, 19, 32^ To further identify whether the apoptotic signaling pathway underlies RanBP9/TSSC3 complex-mediated anoikis, we examined mitochondrial-associated apoptotic proteins. Overexpression of RanBP9 not only reduced Src Tyr^418^, Akt Ser^473^, but also increased cytochrome c in the cytosolic fraction and cleaved caspase-3 in the SaOS2 cells in suspension culture, which were reversed by knockdown of *TSSC3* (Figures 5a and b). Similar trends were observed after overexpression of TSSC3 and knockdown of *RanBP9* (Figures 5a and b). Mitochondrial membrane potential (MMP) is a general indicator of mitochondrial health.^32^ RanBP9-overexpressing SaOS2 cells displayed marked MMP impairment – as indicated by accumulation of monomeric JC-1, which was scored by the ratio of green/red fluorescence intensities of JC-1; however, knockdown of *TSSC3* reversed these effects (Figure 5c; Supplementary Figure S9). Similar effects were observed after overexpression of TSSC3 and knockdown of *RanBP9* (Figure 5c; Supplementary Figure S8). These results indicate that RanBP9/TSSC3 complex facilitates mitochondrial-associated anoikis by downregulating the Src/Akt pathway.

### The RanBP9 SPRY domain is required for RanBP9/TSSC3 complex-mediated anoikis resistance

We hypothesized that RanBP9 SPRY domain is required for RanBP9/TSSC3 complex-mediated anoikis resistance. Exogenous wild-type RanBP9 was interacted with endogenous TSSC3 to exert a tumor-suppressive effect, as indicated by reduced anchorage-independent growth and anoikis resistance, as well as decreased phosphorylations of both Src Tyr^418^ and Akt Ser^473^; however, these tumor-suppressive effects were attenuated by deletion of the SPRY domain of RanBP9 (Figures 5d and f; Supplementary Figure S8b). Moreover, overexpression of RanBP9△SPRY (RanBP9 without SPRY domain) had no effect on the increased anoikis resistance and the phosphorylations of both Src Tyr^418^ and Akt Ser ^473^ induced by knockdown of *TSSC3* (Figures 5g–i; Supplementary Figure S8c). These data suggest that RanBP9 SPRY domain is essential for RanBP9/TSSC3 complex-mediated anoikis resistance in osteosarcoma.

## Discussion

### RanBP9 is a novel TSSC3-interacting protein

The cytoplasmic protein TSSC3 is involved in the cross-talks with multiple critical pathways that regulate tumorigenesis.^9, 10, 11, 12, 13, 14, 15^ We identified a novel structural, functional intracellular interaction between TSSC3 and RanBP9, a scaffolding protein containing multiple conserved domains involved in several signal transduction pathways.^18^ We confirmed both exogenous and endogenous physical interactions between RanBP9 and TSSC3 and determined the binding of TSSC3 PH domain to RanBP9 SPRY domain. The SPRY domain of RanBP9 is reportedly involved in a wide variety of protein–protein interactions.^18, 19^ Deletion of such SPRY domain reduces expression of RanBP9, suggesting that SPRY domain contains important protein folding and/or stability sequences.^19^ The SPRY domain may also regulate the pro-apoptotic activity of RanBP9.^19^ In addition, deletion of SPRY domain reduces the ability of RanBP9 to interact with Mgl-1 so that Mgl-1 has a less inhibitory effect on cell proliferation and migration.^20^ In agreement with these reports, we confirmed that RanBP9 SPRY domain is essential for the anoikis resistance mediated by the physical interaction between RanBP9 and TSSC3.

RanBP9, as a protein stabilizer or a regulator of transcription, has been implicated in various cellular processes that involve both nuclear and cytoplasmic functions.^18^ Post-translational modification of intracellular proteins by ubiquitinating and deubiquitinating enzyme (DUB) regulates multiple cellular processes.^33^ We demonstrated that cytoplasmic RanBP9 and TSSC3 were likely to stabilize each other. Interestingly, RanBP9 is associated with USP11, a DUB that prevents ubiquitination of targeted proteins.^34^ Therefore, we speculate that RanBP9 may promote deubiquitination of TSSC3 by recruiting USP11 to TSSC3. Interestingly, RanBP9 and TSSC3 could also alter the expression of each other at the transcriptional level, and the luciferase reporter promoter assay indicated that RanBP9 and TSSC3 regulate each other at the the transcriptional level. Although primarily localized to the cytoplasm, TSSC3 can enhance transcription by inducing cytoplasmic–nuclear translocation of transcription factors.^35^ Based on these findings, we speculated that nuclear RanBP9 may directly or indirectly interacts with transcription factors to regulate TSSC3 expression and that although primarily localized to the cytoplasm, TSSC3 may enhance RanBP9 transcription by inducing cytoplasmic–nuclear translocation of transcription factors. The transcriptional mechanisms about by which RanBP9 and TSSC3 regulate each other are interesting avenues for our future investigation.

### The Src-dependent Akt pathway is involved in RanBP9/TSSC3 complex-mediated anoikis resistance

**Figure 6:**
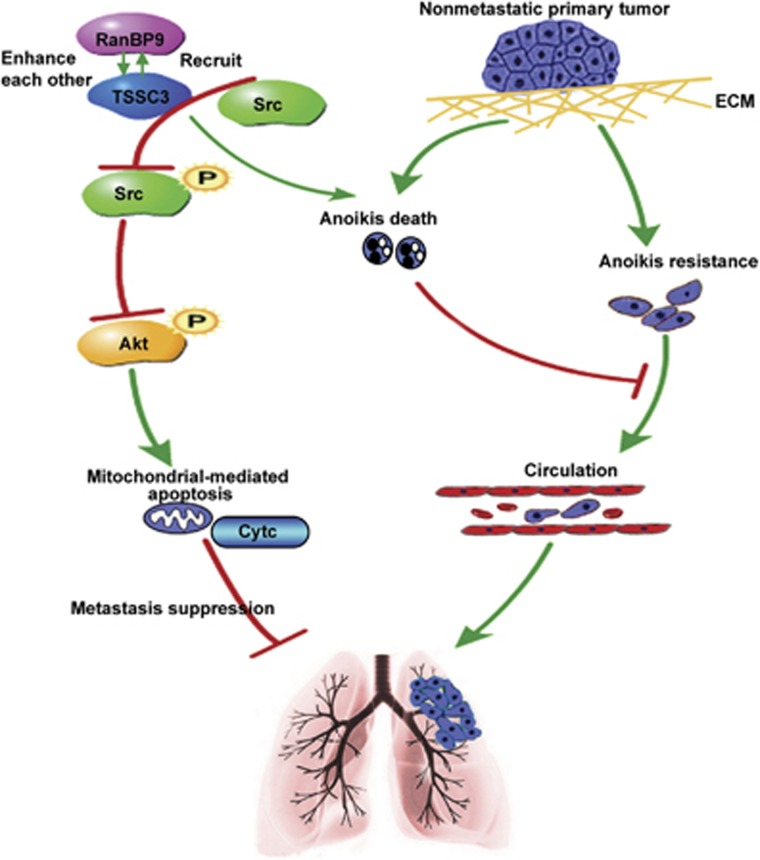
Schematic model to illustrate the anti-metastatic effect of RanBP9/TSSC3 complex in osteosarcoma. RanBP9 and TSSC3 upregulate each other at the transcriptional and post-translational level, and recruit Src to form a ternary RanBP9/TSSC3/Src complex, thereby reducing activation of Src and Akt, which in turn prevents anoikis resistance and metastasis

Src kinase activation exerts a pro-metastatic effect in a variety of tumor types by several key oncogenic mechanisms such as cell proliferation, adhesion, invasion and resistance to apoptosis.^31^ This hypothesis was supported by initial investigations in osteosarcoma of which inhibition of Src activity using the small molecule inhibitor *Dasatinib* inhibited migration and invasion *in vitro* and tumor growth *in vivo*, and restored anoikis sensitivity.^36, 37^ Several independent studies provided further evidence for the role of Src in anoikis resistance in osteosarcoma.^31, 38^ As the proline-rich N-terminus of RanBP9 contains six prototypical SH3-binding domains, RanBP9 was predicted to bind with high affinity to Src.^21^ We hypothesized that Src-dependent Akt pathway may be involved in the function of RanBP9/TSSC3. Our study demonstrates that a ternary RanBP9/TSSC3/Src complex forms in osteosarcoma cells and can be significantly augmented or inhibited by overexpressing or knocking down RanBP9. It is likely that RanBP9 acts by stabilizing TSSC3 and recruiting Src to the RanBP9/TSSC3 complex, thereby reducing Src kinase and Akt activity, and in turn preventing anoikis resistance and metastasis (Figure 6). Thus, we conclude that RanBP9/TSSC3/Src complex acts as an allosteric inhibitor of Src. Indeed, exposure of RanBP9-overexpressing osteosarcoma cells to pYEEI restored Src kinase activity and suppressed anoikis.

TSSC3 is a PH domain-only protein. Thus, by analogy with other PH domain proteins, TSSC3 mostly modulates cell signaling, intracellular trafficking or processes that depend on phosphatidylinositol lipid second messengers.^35^ However, several PH domains are capable of protein–protein interactions, and in most cases, the interacting regions do not overlap with the PH domain lipid-binding region.^39, 40^ A homolog of TSSC3 (PHLDA3) represses Akt activity by competitively binding to PIPs.^41^ Hence, we questioned whether both RanBP9-TSSC3-Src and RanBP9-TSSC3-PIPs complexes regulate Akt. Intriguingly, RanBP9/TSSC3 complex, despite of physical interaction with PIPs, did not prevent Akt from binding to PIPs (data not shown). Although PIP3-binding ability is required for Akt inhibition, not all PH domains that bind PIP3 inhibit Akt, indicating that alternative cooperative mechanisms may exist for selected PH domain functions. In agreement, among proteins possessing PIP3-binding PH domains, Btk and Gab1 inhibit Akt whereas Grp1 and ARNO do not.^40^ Our data demonstrate that RanBP9/TSSC3 complex inhibits the activity of Src and its downstream effectors (PIPs, Akt) by associating with Src, suggesting that RanBP9/TSSC3 complex, at least in osteosarcoma, affects signals upstream of Akt but does not repress Akt activity by competitively binding PIPs.

### Function of RanBP9/TSSC3 complex in osteosarcoma

There is an urgent need to clarify the molecular mechanisms by which metastasis is regulated in osteosarcoma so that novel diagnostic, assessment and therapeutic methods are identified. It has been known that control of pulmonary metastasis is essential to improve prognosis. Downregulation of RanBP9 is associated with the development of cancer.^42^ RanBP9 may function as a tumor suppressor in cervical, colon and lung cancer,^43, 44^ but may act as an oncogene in breast cancer and renal carcinoma.^45, 46^ The role of RanBP9 in tumorigenesis remains controversial because of different findings regarding its ability to activate signaling cascades such as ERK1/2 pathway.^42, 47^ RanBP9 may act as a tissue/cell-specific tumor suppressor depending on its interacting proteins and related regulatory networks. Our study definitely indicates that both RanBP9 and TSSC3 have an essential tumor-suppressor role to prevent metastasis and may represent useful clinical predictors of metastases and/or prognostic factors in osteosarcoma.

We explored whether the inhibitory effects of RanBP9 and TSSC3 on metastasis were due to separate effects of each protein or their function as a complex. Gain-and-loss or loss-and-gain functional approaches confirmed that RanBP9 and TSSC3 functioned cooperatively, likely as a protein complex, to modify anoikis resistance, anchorage-independent growth, and more importantly, the metastatic ability of osteosarcoma cells. Furthermore, we explored the molecular mechanism of the cooperative function of RanBP9/TSSC3 complex. As described above, RanBP9-TSSC3 complex abrogated activation of Src-dependent Akt pathway, which is essential for anoikis resistance. Inhibition of Src increased the sensitivity of osteosarcoma cells to anoikis so that targeting Src may help to eradicate micrometastatic disease and improve prognosis in osteosarcoma.

In summary, this is the first comprehensive study to demonstrate a novel anoikis-promoting, metastasis-suppressive function for RanBP9/TSSC3 complex. RanBP9 functions as a scaffolding molecule to enhance formation of RanBP9/TSSC3/Src complex, which then suppresses activation of Src, negatively regulates Akt pathway and ultimately facilitates mitochondrial-associated anoikis. The functional interaction between RanBP9 and TSSC3 directly regulates anoikis resistance and metastasis, and provides a biological basis for further exploration of the therapeutic significance of dual targeting RanBP9 and TSSC3 in osteosarcoma.

## Materials and methods

### Reagents

The primary antibodies used in this study were as follows: TSSC3 (Abnova, Taipei, Taiwan), RanBP9 (Abcam, Cambrige, MA, USA), cMyc (Santa Cruz Biotechnology, Dallas, TX, USA), Flag (Beyotime, Shanghai, China), ubiquitin (Santa Cruz Biotechnology), Src, Src Tyr418 (Cell Signaling Technology, Danvers, MA, USA), Akt, Akt Ser473, cleaved caspase-3 (Cell Signaling Technology), cytochrome c (Abcam), GAPDH (Cell Signaling Technology), Ezrin (Santa Cruz Biotechnology), BMP, vimentin and CK18 (Zhongshan Golden Bridge Biotechnology, Beijing, China).

Cells were treated with the Src activator pYEEI (Biomol Research Laboratories, Shanghai, China) for 12 h at a final concentration of 100 *μ*mol/l and the Src inhibitor AZD0530 (Saracatinib; www.Selleckchem.com) for 12 h at 3 *μ*mol/l. Cycloheximide (Sigma, St. Louis, MO, USA) was applied for 24 h at a final concentration of 100 *μ*g/ml.

Cytosolic proteins were extracted using the Mitochondria Isolation Kit for Mammalian Cells (Pierce, Rockford, IL, USA) according to the manufacturer's instructions.

The primers used in this study were: RanBP9, 5′-CGCATCCAATACCAGCAGCC-3′ and 5′-GGCACAGTACCCATGGTGGA-3′ TSSC3, 5′-TCCAGCTATGGAAGAAGAAGC-3′ and 5′-GTGGTGACGATGGTGAAGTACA-3′ and GAPDH, 5′-CTTTGGTATCGTGGAAGGACTC-3′ and 5′-GTAGAGGCAGGGATGATGTTCT-3′.

### Cell lines

Immortalized human osteoblast cells hFOB (hFOB1.19) and the osteosarcoma cell lines SaOS2, U2OS and MG63 were obtained from and cultured as described by the American Type Culture Collection (Manassas, VA, USA). The malignant transformed hFOB cell line (MTF cells) was established as previously reported.^8^ Cell lines were used within 6 months of resuscitation of the original cultures.

### Human osteosarcoma specimens

Specimens were obtained from 80 patients with histopathologically confirmed osteosarcoma before radiation therapy or chemotherapy from Xinqiao Hospital and Southwest Hospital, Third Military Medical University (TMMU), Chongqing, China from 2004 to 2011. Detailed clinicopathological features are listed in Table 1. The tumor specimens were independently classified by at least two certified pathologists. Samples containing >50% of RanBP9 or TSSC3-positive cells were designated as 'positive expression'.^48, 49, 50^ Lung metastases and local recurrence were diagnosed by both imaging and pathology, and the surgical margins were classified according to the Enneking staging system. Patients with primary osteosarcoma were classified as with or without metastasis at diagnosis. Written informed consent for the experimental studies was obtained from the patients or their guardians. All experiments were approved by the Institutional Ethics Committee of TMMU.

### Constructs

Human RanBP9 and its variants were subcloned into pcDNA3–6myc (Clontech, Mountain View, CA, USA), human TSSC3 and its variants were subcloned into pCS4–3FLAG (Clontech). Full-length RanBP9 and TSSC3 were also separately cloned into pLenti6.3_MCS (Invitrogen, Grand Island, NY, USA) or pLOV-CMV-eGFP-EF1a-PuroR vector (Neuron Biotech, Shanghai, China). RanBP9-targeting shRNA (target sequence 1: 5′-GGAAUUG-GAUCCUGCGCAU-3′ target sequence 2: 5′-TCTTATCAACAATACCTGC-3′) was inserted into pSUPER.retro.puro vector (Neuron Biotech); the TSSC3-targeting shRNA (target sequence 1: 5′-CCATAGCTGGAAGAGGCTG-3′ target sequence 2: 5′-ATAACTTAAGGCGCCCGTGCA-3′) was inserted into pSilencerTM 5.1 retrovirus (Neuron Biotech). The plasmid or lentiviral vector were transfected into cells as previously described.^14, 15^ To identify interactive domain between RanBP9 and TSSC3, osteosarcoma cells were transfected with the indicated plasmid vector (RanBP9-wide type or TSSC3-wide type or their variants), and in all the other tests the lentiviral vectors were applied.

### Yeast two-hybrid analysis

The yeast two-hybrid Matchmaker GAL4 Two-Hybrid System 3 (Clontech) was used to identify potential binding partners for TSSC3. Full-length human TSSC3 (Genbank, NP_003302) cDNA was cloned into modified pGBKT7 vector containing the DNA-binding domain and was used as bait to screen the human fetal brain Matchmaker cDNA Library (Clontech). The human fetal brain cDNA library and pGBKT7-TSSC3 were co-transformed into yeast strain AH109 and cultured in yeast drop-out minimal media lacking tryptophan and leucine and containing X-gal. Positive clones were picked for nucleotide sequencing. Sequencing and BLAST searches identified RanBP9 (NM_005493.2) as a putative binding protein for TSSC3. To confirm the interaction between TSSC3 and RanBP9, we co-transformed constructs expressing TSSC3 and RanBP9, or positive and negative controls, into yeast strain AH109 and plated the cells on yeast drop-out minimal media lacking tryptophan and leucine, and assayed for galactosidase activity. Positive clones were sequenced and compared with reference sequences available in GenBank.

### Quantitative real-time PCR analysis, western blot analysis and co-immunoprecipitation

Quantitative real-time PCR analysis and western blotting were performed exactly as previously described.^14^ Co-immunoprecipitations of whole-cell lysates were performed using anti-TSSC3, anti-RanBP9, anti-cMyc, anti-Flag, anti-ubiquitin, anti-Src or nonspecific IgG antibodies using the Dynabeads Co-Immunoprecipitation Kit (Invitrogen) according to the manufacturer's protocol. Pre-immunoprecipitated input samples (10% input) were subjected to western blotting to confirm antibody specificity.

### Luciferase reporter assay

The human RanBP9 promoter or TSSC3 promoter (upstream 2000 to 0) in pGL3 basic plasmids (Promega, Madison, WI, USA) were obtained with the assistance of Invitrogen Company (Shanghai, China). The open reading frame (ORF) of TSSC3 was amplified by PCR as previously described ^14^ and ORF of RanBP9 was obtained from Invitrogen Company (Karlsruhe, Germany). Reporter assays were performed using 250 ng of human RanBP9 or TSSC3 promoter. SaOS2 cells were co-transfected with 1 ng of plamids as indicated (including overTSSC3, siTSSC3, overRanBP9 or siRanBP9). SV-40-Renilla luciferase plasmid (1 ng) was co-transfected to control the efficiency of transfection. Expression of Firefly and Renilla luciferases were analyzed 48 h post-transfection by using a luciferase assay kit (Promega), according to the manufacturer's instructions.

### Immunofluorescence, histology, immunohistochemistry and TUNEL assay

Assay was performed as previously described.^14^

### Soft agar assay, Annexin V/7AAD analysis, mitochondrial membrane potential analysis and Src tyrosine kinase activity assay

Anchorage-independent growth was assessed using the colony formation assay in soft agar on 0.35% low melting-point agar (Invitrogen) overlaid on 0.6% agarose. Cell suspensions (3 × 104 cells per 60 mm dish) were plated in semisolid medium (DMEM containing 10% FBS plus 0.35% agar) and incubated at 37 °C in a humidified 5% CO_2_ atmosphere. Colonies were counted after 10 days. Annexin V/7AAD analysis and mitochondrial membrane potential analysis were performed as described previously.^14^ Briefly, Annexin V/7AAD analysis was performed by using an Annexin V-APC antibody (eBioscience, Santiago, CA, USA) and 7AAD antibody (KeyGEN, Nanjing, China). Harvested cells were washed three times in PBS buffer and then resuspended in 250 *μ*l binding buffer (eBioscience). Subsequently, a total of 8 *μ*l of 7AAD and 5 *μ*l of Annexin V-APC were added and incubated for 10 min. Then, the stained cells were analyzed by flow cytometry (10 000 cells; FACSCalibur; Becton Dickinson, Franklin lakes, NJ, USA). Src tyrosine kinase activity was detected using the Src Assay Kit (Millipore, Plano, TX, USA) following the manufacturer's instructions.

### *In vivo* lung metastasis model

Animal care and experimental procedures were conformed to the Institutional Animal Care and Use Committee of Southwest Hospital, TMMU according to the Guide for the Care Use of Laboratory Animals. Single-cell suspensions of 7 × 10^6^ cells/0.5 ml were injected into the tail vein of 6-week-old female severe combined immunodeficient mice (Laboratory Animal Center, Southwest Hospital, TMMU). The mice were evaluated daily for emaciation, lethargy or other signs of incapacitating tumor burden. The mice were killed 63 days after injection and the lungs were resected and fixed in formalin, and the visible tumor nodules (macrometastases) were counted. The fixed lungs were then embedded in paraffin, sectioned, and stained with hematoxylin and eosin, and microscopic lung metastasis were counted under a light microscope.

### Statistical analysis

All values are presented as mean±S.D. Data were analyzed using Student's *t*-test, Spearman rank correlation coefficients or Pearson *χ*^2^ test. Survival analysis was carried out by using the log-rank test. *P*<0.05 was considered statistically significant. All analyses were performed using SPSS 19.0 software (version 19.0; SPSS Inc., Chicago, IL, USA).

## Figures and Tables

**Table 1:** Correlations between RanBP9 and TSSC3 expression and the clinicopathological features of osteosarcoma

			**RanBP9**			**TSSC3**		
**Clinical characteristics**	**Group**	***n***	**Positive**	**Negative**	***P*-value**^a^	**Positive**	**Negative**	***P*-value**^a^
Age (years)	11–20	34	27	7	0.182	25	9	0.407
	21–30	23	21	2		19	4	
	≥31	23	16	7		15	8	
Gender	Male	53	41	12	0.408	37	16	0.262
	Female	27	23	4		22	5	
Tumor location	Limbs	70	57	13	0.398	54	16	0.068
	Others	10	7	3		5	5	
Stage	I	6	6	0	0.001^b^	6	0	0.004^b^
	II	61	55	6		48	13	
	III	13	3	10		5	8	
Lung metastasis	Yes	13	3	10	<0.001^b^	5	8	0.002^b^
	No	67	61	6		54	13	
Local recurrence	Yes	15	7	8	<0.001^b^	7	8	0.008^b^
	No	65	57	8		52	13	
Grade	Low	6	6	0	0.203	6	0	0.129
	High	74	58	16		53	21	
Histological type	Osteoblastic	28	19	9	0.083	21	7	0.824
	Fibroblastic	4	3	1		3	1	
	Chondroblastic	32	26	6		22	10	
	Others	16	16	0		13	3	
Tumor size	<10 cm	65	52	13	1.000	46	19	0.207
	≥10 cm	15	12	3		13	2	

a Pearson's *χ*^2^ test

b With significant difference
