# Supplementary material for: RanBP9/TSSC3 complex cooperates to suppress anoikis resistance and metastasis via inhibiting Src-mediated Akt signaling in osteosarcoma
Source: Cell Death Dis. 2016 Dec 29;7(12):e2572–. doi: 10.1038/cddis.2016.436 (PMC5261021; doi:10.1038/cddis.2016.436)
Supplement: Supplementary Information [file cddis2016436x1.doc]

**Supplementary Table**

**Table S1. Results of Yeast Two-Hybrid Analysis**

| Bait | Bait Prey  library | Identical  colonies | positive gene  identified | NCBI accession  number | Gene  Code  match |
| --- | --- | --- | --- | --- | --- |
| PGB-  TSSC3 | Human Fetal Brain  MATCHMA KER cDNA Library (Clontech, CAT#HL4028AH) | 5 | RanBP9 | NM_  005493.2 | 5 |

**Table S2.** Correlation between RanBP9 and TSSC3 expression in human osteosarcoma

| RanBP9 expression | TSSC3 expression  Positive | Negative |
| --- | --- | --- |
| Positive | 57/64 | 7/64 |
| Negative | 2/16 | 14/16 |
| *P*-value**^＃^** | <0.001* |  |

**^＃^**Spearman’s rank correlation coefficient

^＊^with significant difference

**Table S3.** Expression of Ezrin positively correlates with the presence of lung metastasis in human osteosarcoma

| Ezrin expression | Lung metastasis  Yes | No |
| --- | --- | --- |
| Positive | 12/13 | 1/13 |
| Negative | 14/67 | 53/67 |
| *P*-value**^＃^** | <0.001* |  |

**^＃^** Pearson’s χ^2^ test

^＊^with significant difference

**Table S4.** Expression of RanBP9 and TSSC3 correlate negatively with expression of Ezrin in human osteosarcoma

| Ezrin expression | TSSC3  Positive | expression  Negative | RanBP9  Positive | expression  Negative |
| --- | --- | --- | --- | --- |
| Positive | 8/26 | 18/26 | 13/26 | 13/26 |
| Negative | 51/54 | 3/54 | 51/54 | 3/54 |
| *P*-value**^＃^** | <0.001* |  | 0.001* |  |

**^＃^**Spearman’s rank correlation coefficient

^＊^with significant difference

**Supplementary figure legends**

**Figure S1.** RanBP9 interacts with TSSC3 transcriptionally.

**(a-b)**,TSSC3 and RanBP9 transcriptionally regulate each other. Quantitative RT-PCR analysis of *TSSC3* in the indicated RanBP9-overexpressing/-knockdown cells (a) and *RanBP9* in TSSC3-overexpressing/-knockdown cells (b). Gene expression was normalized to *GAPDH* and expressed as fold change relative to controls. Values are mean ± SD for one experiment (*n* = 4) representative of three independent experiments; *** *P* < 0.001.

**(c)**, Influence of overRanBP9 and siRanBP9 on the luciferase activity of the TSSC3-promoter constructs (left) or overTSSC3 and siTSSC3 on the luciferase activity of the RanBP9-promoter constructs (right). The recombinant constructs as indicated were separately transfected into SaOS2 cells, and cells were harvested 48 h later for dual luciferase activity assays.

**Figure S2.** The expression of RanBP9 or TSSC3 in osteosarcoma cell lines.

**(a-b)**, Quantitative RT-PCR analysis (i) and Western blot analysis (ii) of RanBP9 and TSSC3 in MTF cells and hFOB1.19 osteoblasts (a) and the highly metastatic cell lines (SaOS2, U2OS, MTF) and less-metastatic cell line MG63 (b). Gene expression was normalized to *GAPDH* and expressed as fold change relative to controls. Values are mean ± SD for one experiment (*n* = 4) representative of three independent experiments; *** *P* < 0.001.

**(c)** Immunofluorescent analysis of RanBP9 (red) and TSSC3 (green) in osteosarcoma cell lines, scale bars = 50 µm.

**Figure S3.** Loss of RanBP9 and TSSC3 promotes a highly anoikis-resistant phenotype in osteosarcoma cell lines

**(a)** Suspended parental SaOS2 cells undergo anoikis in a time-dependent manner. SaOS2 cells cultured under adherent conditions (adhered) were trypsinised and cultured in ultralow attachment 6 well plates (suspended). After the indicated times, the cells were harvested and subjected to flow cytometric analysis.

**(b)** Morphology of SaOS2 aggregates that survived culture in ultralow attachment 6 well plates at the indicated times viewed under the light microscope (photographed at ×40 magnification).

**(c-d)** RanBP9 and TSSC3 are downregulated in cells that survive induction of anoikis. SaOS2 and MG63 cells cultured under adherent conditions (adhered) were seeded into ultralow attachment 6 well plates for suspension culture (suspended). After the indicated times, anoikis-resistant cells (AnnexinV-APC—/7AAD— cells) were sorted by flow cytometry. c, Quantitative RT-PCR analysis of RanBP9 and TSSC3. Gene expression was normalized to GAPDH and expressed relative to 0 day. Values are mean ± SD for one experiment (*n* = 4) representative of three independent experiments; **P* < 0.05, ***P* < 0.01, ****P* < 0.001, NS: no significance. d, Western blot analysis of RanBP9 and TSSC3. Western blot values were normalized to GAPDH.

**Figure S4.** Representative images of immunohistochemical analysis of the expression of RanBP9 and TSSC3 in human osteosarcoma samples with and without metastasis. Scale bars: 50 μm.

**Figure S5.** Western blot analysis of RanBP9 and TSSC3. SaOS2 **(a)**, MG63 **(b)** and MTF **(c)** cells were stably transfected as indicated, and the expression of RanBP9 and TSSC3 were confirmed by Western blot analysis. Western blot values were normalized to GAPDH.

**Figure S6.** RanBP9 and TSSC3 function cooperatively to inhibit apoptosis under adherent culture condition. SaOS2 or MG63 cells were stably transfected with the indicated constructs, then were cultured under adherent culture conditions. **(a)** TUNEL assay of SaOS2 (left) or MG63 (right) cells observed by laser confocal scanning microscopy. Green FITC-positive cells represented apoptotic cells. Scale bars: 50 μm. (b) Western blot analysis of cleaved caspase-3 expression in SaOS2 (left) or MG63 (right) cells. Western blot values were normalized to GAPDH.

**Figure S7.** Immunocytochemical analysis of BMP, Vimentin and CK18 in metastatic lung nodules from the in vivo metastasis model.

**Figure S8.** RanBP9 modulating anoikis resistance is dependent on Src kinase activity and the RanBP9 SPRY domain.

Representative flow cytometric analyses of apoptosis were shown. **(a)** RanBP9 overexpression or knockdown SaOS2 cells were cultured in suspension in the presence or absence of Src activator pYEEI (100 µmol/L) or Src inhibitor AZD0530 (3 µmol/L). **(b)** SaOS2 cells transfected with MOCK, RanBP9-WT or RanBP9△SRRY were cultured in suspension. **(c**) SaOS2 cells co-transfected with the TSSC3si/NCsi together with wild-type RanBP9 or RanBP9△SPRY were cultured in suspension. **(c**) SaOS2 cells co-transfected with the TSSC3si/NCsi together with wild-type RanBP9 or RanBP9△SPRY were cultured in suspension.

**Figure S9.** Mitochondrial transmembrane potential assay. Representative photographs from positive control (PC) and the indicated groups. Green fluorescence represented the monomeric form of JC-1. Red fluorescence represented the mitochondrial aggregate form of JC-1. Scale bar =50µm.
