# Supplementary figures and images for: RanBP9/TSSC3 complex cooperates to suppress anoikis resistance and metastasis via inhibiting Src-mediated Akt signaling in osteosarcoma
Source: Cell Death Dis. 2016 Dec 29;7(12):e2572–. doi: 10.1038/cddis.2016.436 (PMC5261021; doi:10.1038/cddis.2016.436)

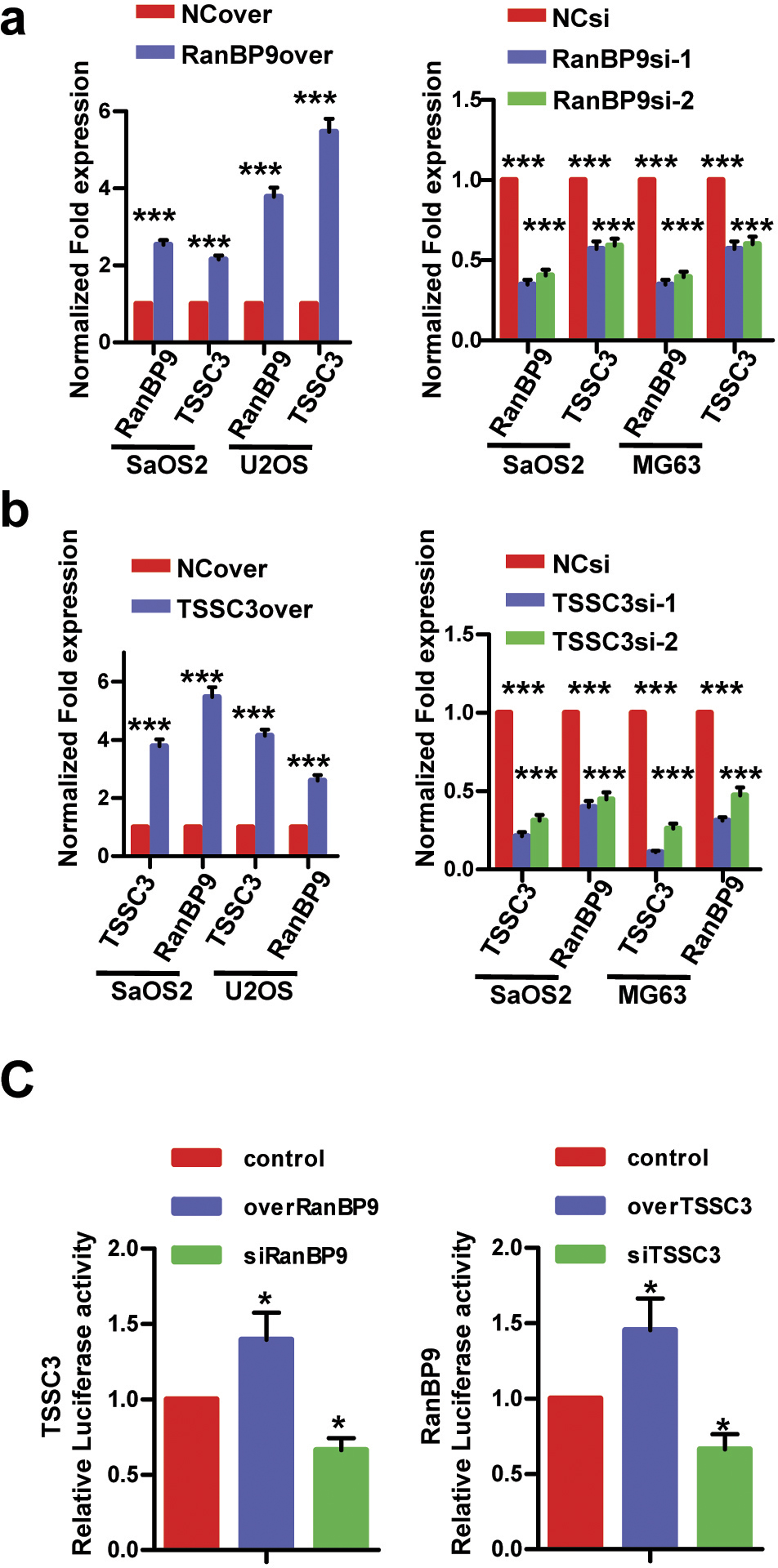

Supplement: Supplementary Figure 1 [file cddis2016436x2.tif]

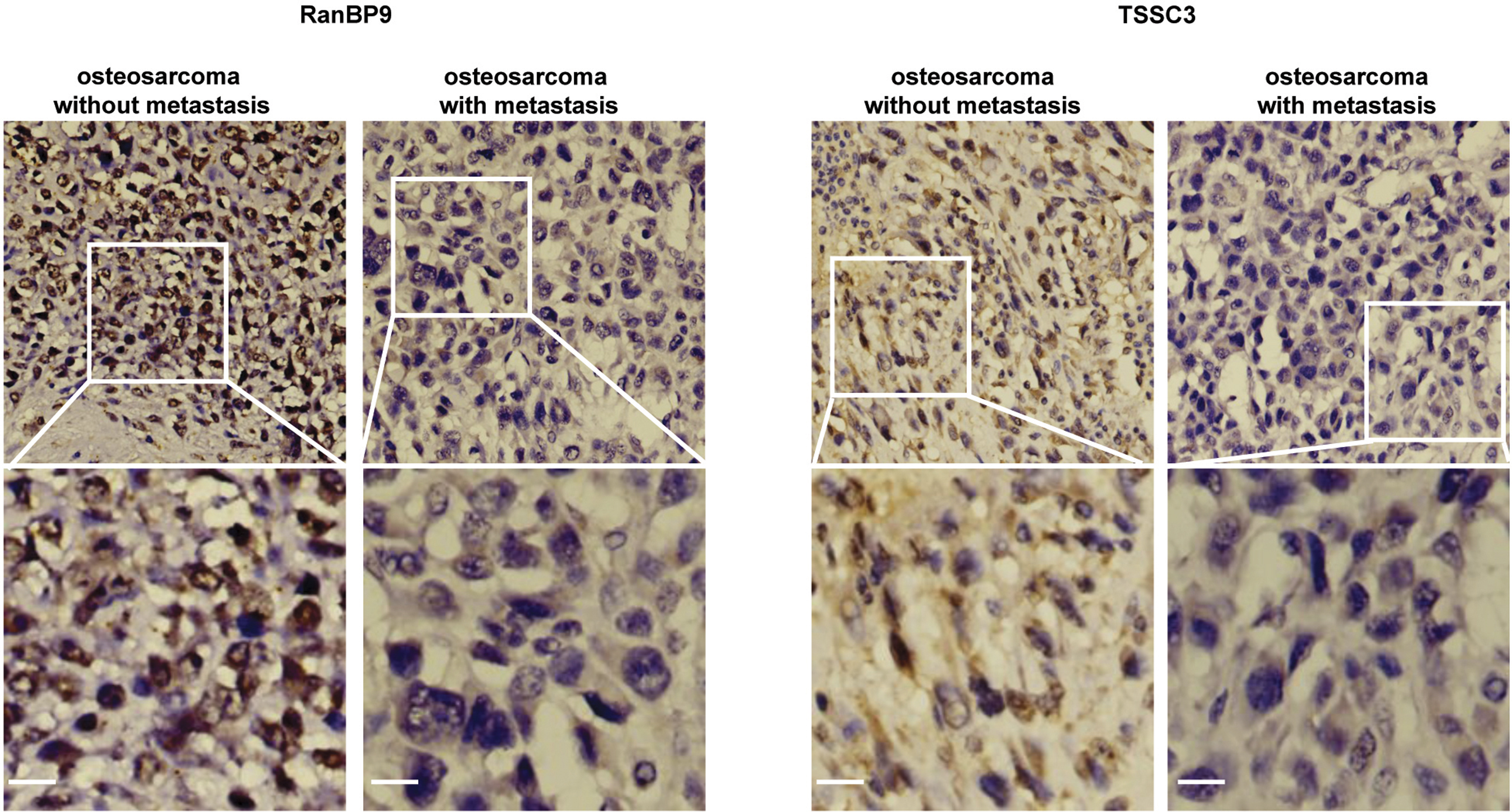

Supplement: Supplementary Figure 4 [file cddis2016436x5.tif]

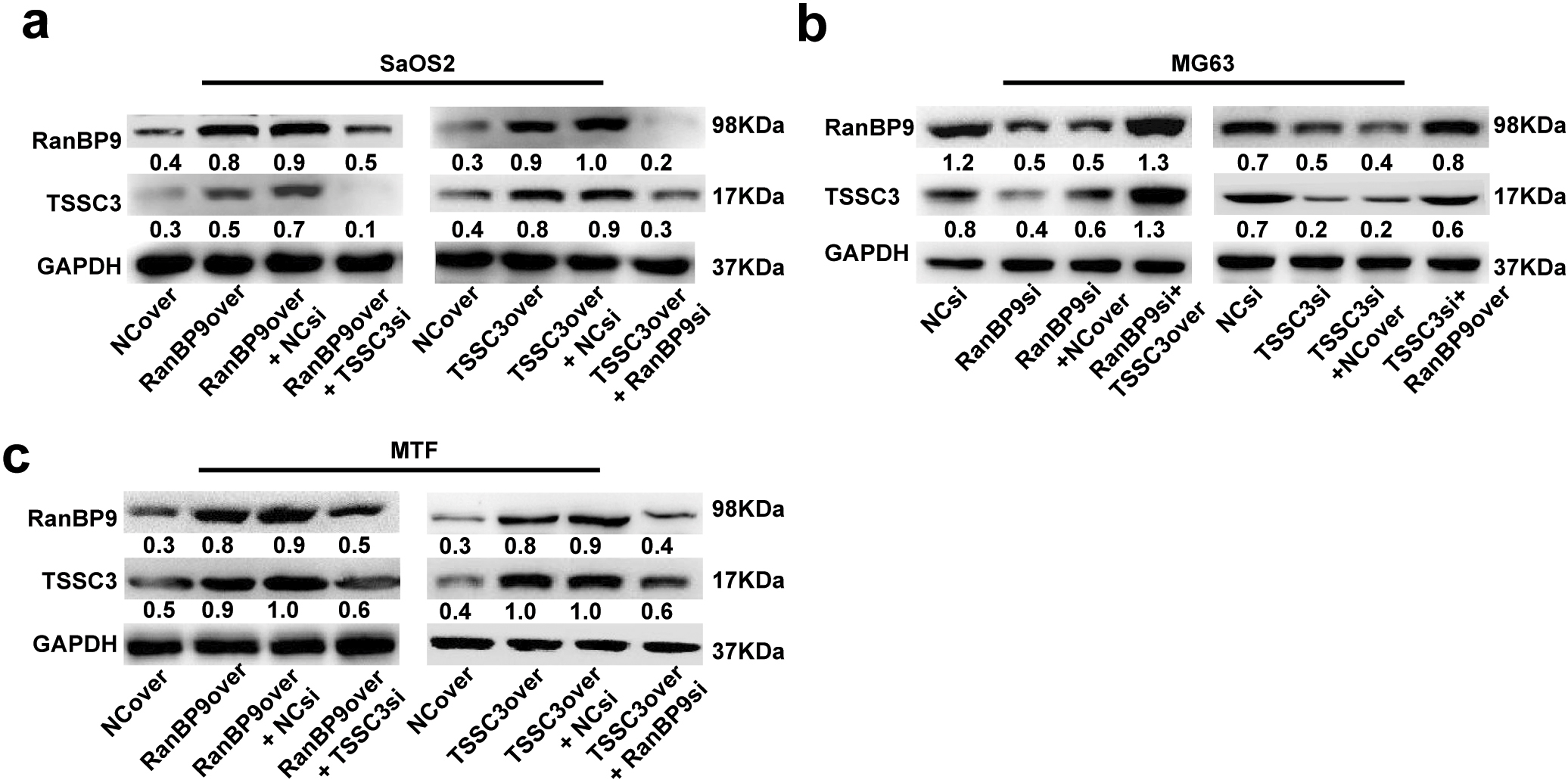

Supplement: Supplementary Figure 5 [file cddis2016436x6.tif]

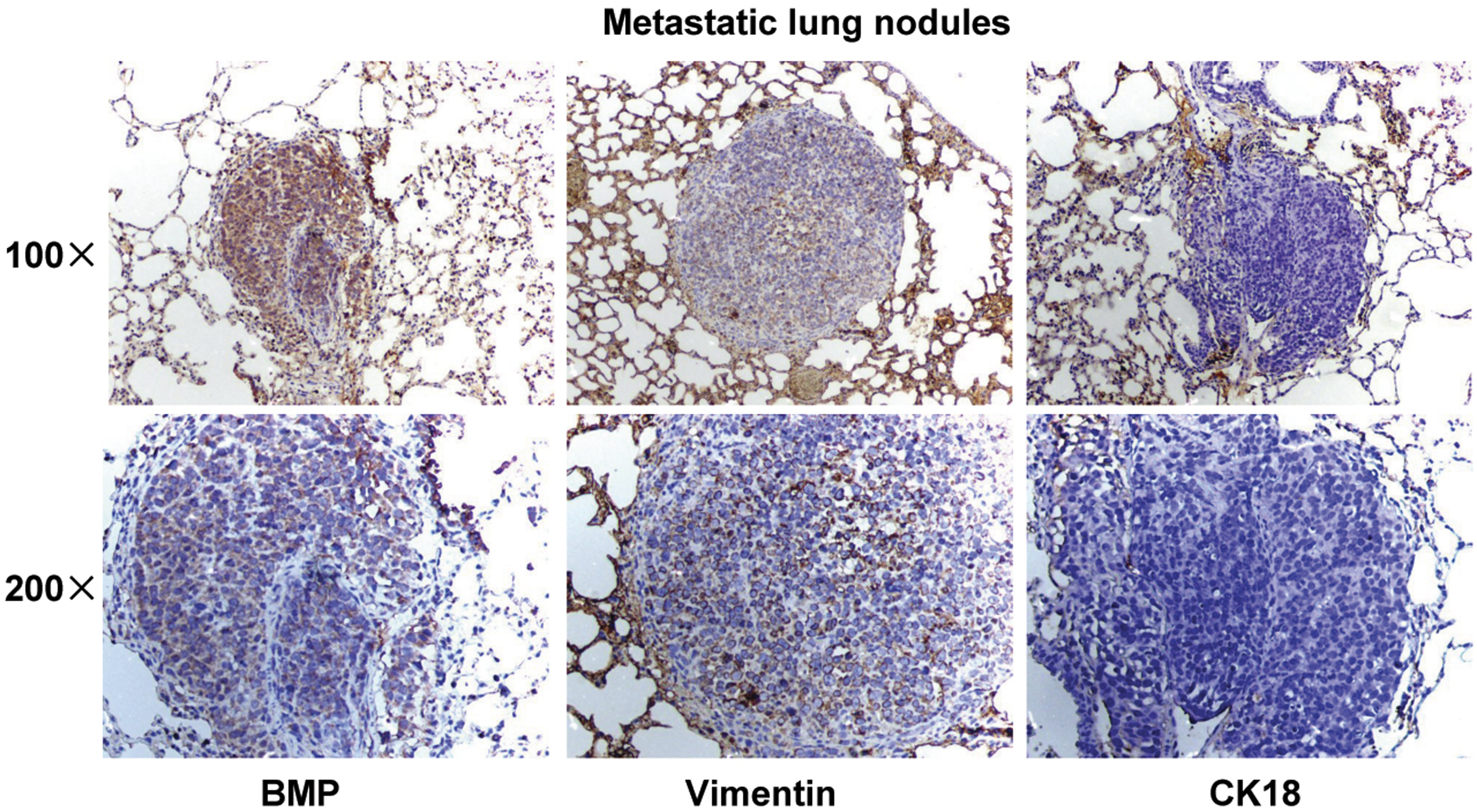

Supplement: Supplementary Figure 7 [file cddis2016436x8.tif]

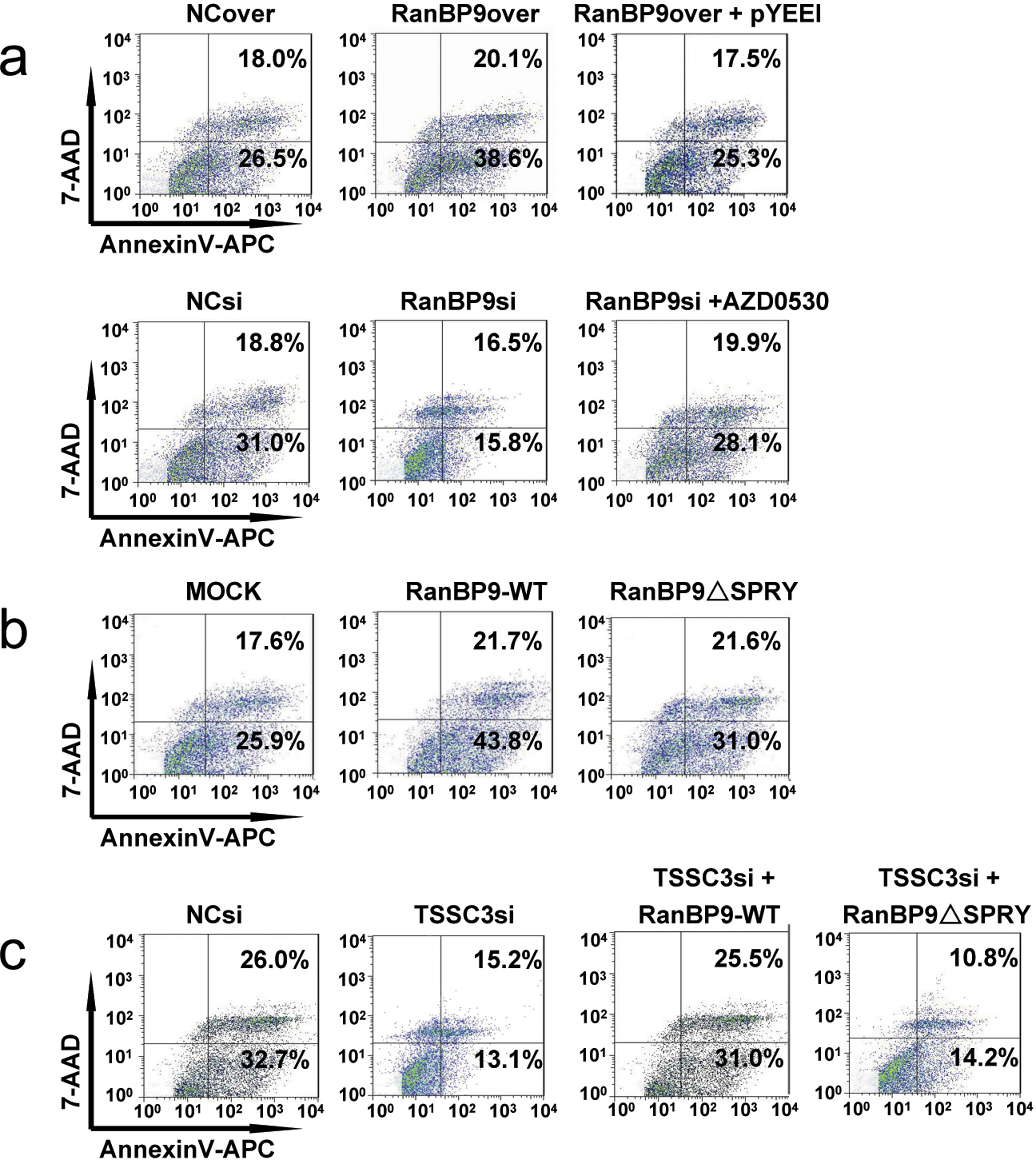

Supplement: Supplementary Figure 8 [file cddis2016436x9.tif]

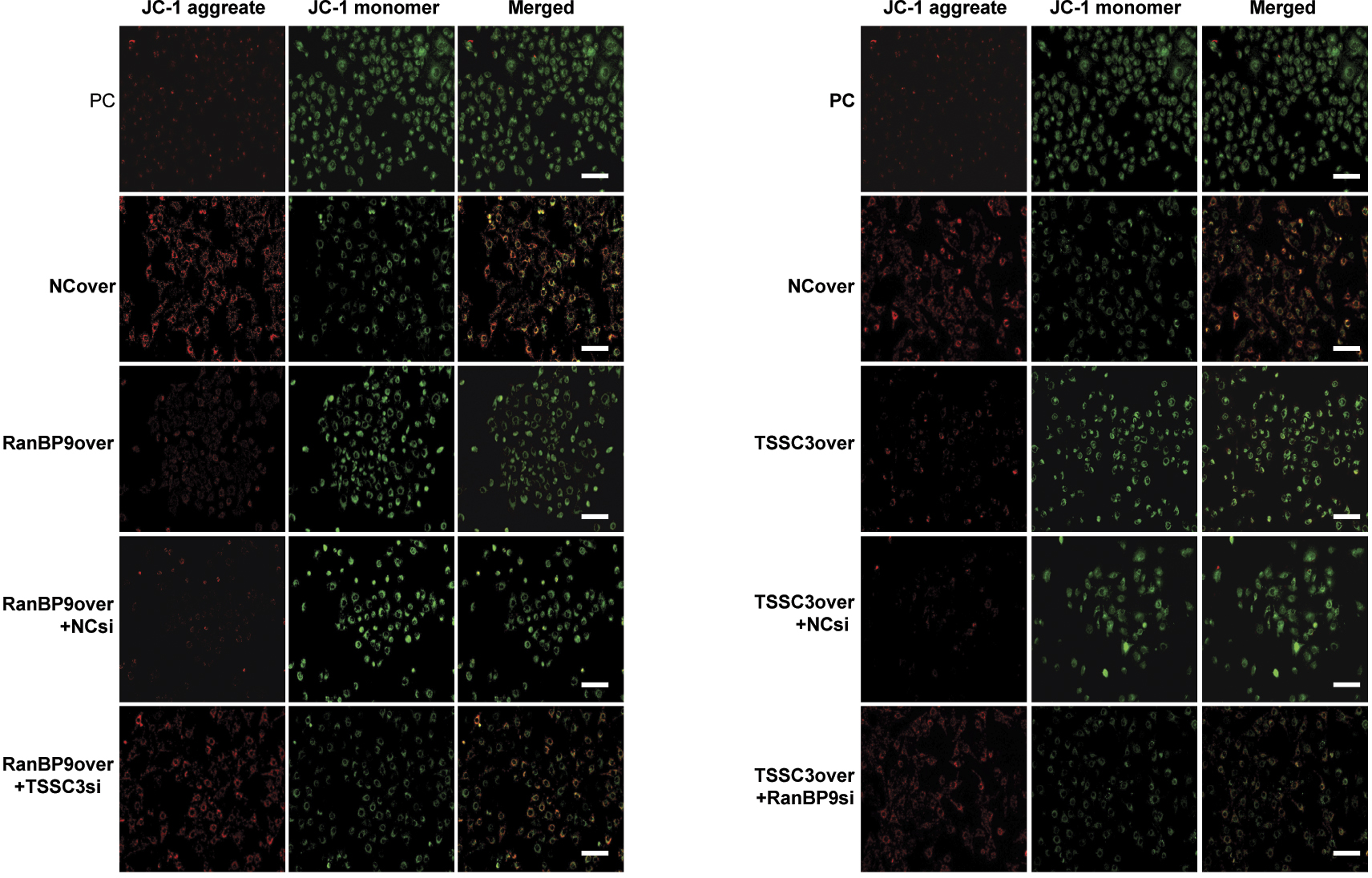

Supplement: Supplementary Figure 9 [file cddis2016436x10.tif]
